# Harsh Childhood Discipline and Developmental Changes in Adolescent Aggressive Behavior: The Mediating Role of Self-Compassion

**DOI:** 10.3390/bs13090725

**Published:** 2023-08-30

**Authors:** Qing-Feng Yang, Rui-Bo Xie, Rui Zhang, Wan Ding

**Affiliations:** Parent Education Research Center, Intelligent Laboratory of Child and Adolescent Mental Health and Crisis Intervention of Zhejiang Province, School of Psychology, Zhejiang Normal University, Jinhua 321004, China

**Keywords:** psychological aggression, corporal punishment, self-compassion, aggressive behavior, developmental trajectories

## Abstract

Harsh discipline during childhood (psychological aggression and corporal punishment) has been found to be an early risk factor for adolescent aggressive behavior. However, previous studies have mainly examined the relationship between harsh discipline as a whole and the level of adolescent aggressive behavior. This study investigates the effects of childhood psychological aggression and corporal punishment on the initial levels and rate of change in adolescent aggressive behavior, as well as the mediating role of self-compassion in this relationship. Using cluster sampling, a three-wave follow-up assessment was conducted on 1214 high-school students (60.7% boys; mean age at Wave 1 = 15.46 ± 0.71). The results showed that childhood psychological aggression and corporal punishment had a positive predictive effect on the development level of adolescent aggressive behavior. However, only childhood psychological aggression significantly directly attenuated the decline rate of adolescent aggressive behavior. In addition, both childhood psychological aggression and corporal punishment indirectly affected the initial levels and growth rate of adolescent aggressive behavior through self-compassion. These findings could provide potential targets for prevention and intervention programs aimed at improving aggressive behavior in Chinese adolescents.

## 1. Introduction

Adolescent aggressive behavior is an important social issue that requires attention globally. It can be defined as any behavior directed towards oneself or others with the intention of causing harm [1]. A recent report by the World Health Organization (WHO) estimated that four out of ten young people aged 10 to 29 had engaged in physical conflicts with others in the year prior to the study, and approximately one-fourth of adolescents experienced bullying in the past month [2]. Additionally, schools may be the most common setting for adolescent aggressive behavior, which includes verbal abuse, physical fights, and self-harm, posing serious threats to students’ physical and psychological well-being and school safety [3,4]. Previous research has shown that adolescent aggressive behavior can lead to irreparable harm for both the aggressors and victims, including emotional distress, academic disengagement, social difficulties [5,6], and even future criminal behavior that is closely associated with aggressive behavior during this period [7]. Therefore, gaining a deeper understanding of adolescent aggressive behavior and exploring the mechanisms of its risk factors are of great significance for the positive development of adolescents. This study aims to explore the relationship between childhood harsh discipline and the development of adolescent aggressive behavior, as well as the potential mediating role of self-compassion in this association.

Adolescence is a critical period for the development of aggressive behavior [8], which can be manifested through both the initial level and the rate of change. The social–cognitive information-processing model of aggression [9] suggests that harsh and punitive parenting styles by parents are important risk factors for adolescents’ adoption of aggression as a problem-solving strategy. Therefore, the developmental changes in adolescent aggressive behavior may be related to the experiences of harsh parenting during childhood. Harsh discipline is a common form of negative parenting during childhood, including harsh psychological aggression and corporal punishment [10]. Previous research has found that parents often employ harsh parenting styles during childhood to teach social norms and correct undesirable behavior [11]. However, adults who have experienced harsh discipline during childhood have been found to exhibit more external aggressive behavior in daily life [12]. It is worth noting that only a few studies have explained the relationship between harsh discipline and the level of adolescent aggressive behavior development [13], and there has been limited attention to the impact of harsh discipline on the rate of change in adolescent aggressive behavior. Moreover, existing research on the relationship between parental harsh discipline and children’s aggressive behavior has mainly focused on corporal punishment or overall harsh discipline, while little attention has been paid to the unique effects of two common types of harsh discipline, psychological aggression and corporal punishment, and the underlying mechanisms between the two and adolescent aggression.

### 1.1. Relationship between Psychological Aggression/Corporal Punishment during Childhood and Developmental Changes in Adolescent Aggressive Behavior

Adolescence is often referred to as a period of “storm and stress”, characterized by heightened fluctuations in emotions and interpersonal distress [14], as well as a heightened desire for sensation seeking and risk taking [15]. Meanwhile, the brain mechanisms and neural circuits associated with aggressive behavior are in a state of developmental mismatch or imbalance during this period, which may make it difficult for adolescents to regulate and inhibit their aggressive impulses, increasing the risk of engagement in aggressive behavior [16]. Although previous research indicates that the majority of adolescents show an overall decline in aggressive behavior as they age [17,18], there is still a subset of adolescents who exhibit persistently high levels of aggression [17,19]. Moreover, considering that the consequences of aggressive behavior often accumulate and may have long-lasting negative effects on subsequent outcomes [20,21], further exploration of risk factors that contribute to the development of adolescent aggressive behavior is necessary. Additionally, there are distinctions between the initial level and developmental trajectories of aggressive behavior [22]. The former reflects both the development of adolescent aggression and significantly predicts future levels of aggression [23], whereas the latter reflects the rate of change in adolescent aggressive behavior. Therefore, examining both the initial level and the rate of change in adolescent aggressive behavior is conducive to further revealing the pattern of change in adolescent aggressive behavior and the role of risk factors in the developmental changes in adolescent aggressive behavior.

In recent years, there has been increasing attention on the impact of harsh discipline on the development of aggressive behavior in children and adolescents [24,25]. Researchers have distinguished between psychological aggression and corporal punishment within harsh discipline. Psychological aggression refers to the use of language and symbolic behaviors by parents to cause psychological pain or fear in children, while corporal punishment involves the use of physical force to make children experience pain, without the intention to cause deliberate harm [26], both of which are widely used by parents in childhood. The social–cognitive information-processing model of aggression suggests that both types of harsh discipline may provide children with negative examples of physical and verbal aggression, making them more likely to adopt the same strategies to cope with emotions or interpersonal problems in the future. Furthermore, various forms of corporal punishment have been found to be risk factors for different stages of aggressive behavior [27,28]. In contrast, previous research has paid less attention to the relationship between psychological aggression and the development of aggressive behavior. Compared to physical violence, psychological aggression is more common and hidden, and some parents may not even be aware of the potential threats to their children’s future development posed by this parenting style [11,29]. However, similar to corporal punishment, parental psychological aggression may also increase children’s aggressive behavior [25,30]. Therefore, it is necessary to comprehensively examine the potential adverse effects of childhood psychological aggression and corporal punishment on the development of adolescent aggressive behavior.

Additionally, there is limited evidence indicating a relationship between harsh discipline and the initial level and rate of change in adolescent aggressive behavior. Research with children by Prinzie et al. [31] suggests that higher levels of coercive parenting behaviors by parents are associated with higher initial levels of child aggressive behavior and slower declines. Moreover, Baydar and Akcinar [32] found that harsh parenting at age 4 positively predicts the developmental trajectory of aggressive behavior in children aged 4–7. These two studies are most relevant to the research question proposed in this study, but neither of them distinguishes between psychological aggression and corporal punishment, and Baydar and Akcinar only examined the parenting styles of mothers. Furthermore, the developmental characteristics of aggressive behavior during childhood and adolescence may differ [33]. In contrast, this study will comprehensively examine the impact of experiencing different types of harsh discipline during childhood on the initial level and rate of change of aggressive behavior during adolescence. Based on the literature mentioned above, we propose the following hypotheses:

**Hypothesis** **1.** *Aggressive behavior continues to develop during adolescence and shows a declining trend*.

**Hypothesis** **2.** *Parental psychological aggression and corporal punishment in children positively and directly predict the initial level of adolescent aggressive behavior*.

**Hypothesis** **3.** *Parental psychological aggression and corporal punishment in children negatively predict the rate of change of adolescent aggressive behavior*.

### 1.2. The Mediating Role of Self-Compassion

The general aggression model [34,35] and recent research suggest that self-compassion may serve as a potential mediator between harsh discipline during childhood and the development of aggressive behavior in adolescence [13]. In this model, negative parenting styles experienced in childhood serve as distal environmental factors that increase the likelihood of engaging in aggressive behavior by interfering with an individual’s internal states (i.e., cognition, affect, and arousal) [34]. Self-compassion refers to adopting a supportive, connected, and present attitude when experiencing failure, inadequacy, and suffering [36]. This adaptive cognitive–emotional regulation strategy consists of three relative components: self-kindness rather than self-judgment, mindfulness rather than overidentification, and recognition that one’s pain and struggles are part of shared humanity rather than isolated experiences [37].

Firstly, childhood psychological aggression and corporal punishment may impair adolescent self-compassion. Evidence exploring the developmental pathways of self-compassion suggests that self-compassion stems from positive interactions with caregivers in childhood (for reviews, see [36,38]). Specifically, parents who express care and warmth may guide children in forming positive beliefs about themselves and their relationships with others, leading to a tendency to provide support for themselves and others [39]. Conversely, children who are chronically exposed to low-warmth, high-rejection parenting environments are more likely to perceive the external environment as hostile and unsafe and view themselves and others negatively, thus making it difficult for them to develop self-compassion [40]. Once such internal processing patterns are formed during childhood, they may persist into adolescence and influence the development of aggressive behavior [38].

In addition, self-compassion may reduce the occurrence and development of adolescent aggressive behavior. Previous research in adult samples has shown that individuals with self-compassion are more accepting of their own and others’ flaws, exhibit more positive interpersonal behaviors, and display less detachment, control, and verbal or physical aggression, compared to individuals lacking self-compassion [13,41,42]. However, representative evidence for adolescents is lacking in such studies. Moreover, there is a lack of focus on the development trajectory of aggressive behavior rather than its level of development. Adolescence is a unique period of self-awareness development, during which individuals become increasingly concerned about their own successes and setbacks, and their understanding of these events may directly influence their coping strategies with themselves and others [43]. Therefore, it is necessary to enhance understanding of the role of self-compassion in reducing the initial level and rate of development of adolescent aggressive behavior.

In conclusion, we have reason to believe that self-compassion may mediate the relationship between harsh discipline during childhood and the development changes of adolescent aggressive behavior. However, this hypothesis has received limited attention and it is not yet clear how different forms of harsh discipline are linked to the initial level and rate of development of adolescent aggressive behavior through self-compassion. Based on the above theory and the findings of previous studies, we propose the following hypotheses:

**Hypothesis** **4.** *Self-compassion mediates the relationship between psychological aggression during childhood and the initial level/development rate of adolescent aggressive behavior*.

**Hypothesis** **5.** *Self-compassion mediates the relationship between corporal punishment during childhood and the initial level/development rate of adolescent aggressive behavior*.

### 1.3. Current Study

This study aims to further explore the relationship between harsh discipline during childhood and the development of adolescent aggressive behavior and to reveal the potential mechanisms underlying this relationship. The main research objectives include: (1) exploring the developmental changes of adolescent aggressive behavior; (2) simultaneously examining the impact of psychological aggression and corporal punishment during childhood on the initial level and rate of change of adolescent aggressive behavior; (3) investigating the mediating role of self-pity in the relationship between psychological aggression and corporal punishment during childhood and the initial level and rate of change of adolescent aggressive behavior. Considering that adolescent gender, age, and SES may have an impact on adolescent aggressive behavior [34,44], this study will control for these variables in subsequent analyses.

## 2. Materials and Methods

### 2.1. Participants

Using a cluster sampling method, high-school students from an urban middle school in an eastern province of China were selected as the participants for this study. Three consecutive questionnaire surveys were conducted with a 6-month interval. A total of 1214 questionnaires were distributed in the first test, with the average age of the participants being 15.46 ± 0.71 years and 60.7% being male. Due to transfers and leaves, 1037 and 964 questionnaires were collected in the second and third tests, respectively.

### 2.2. Measurements

#### 2.2.1. Harsh Discipline during Childhood

The parental conflict tactics scale, developed by Straus et al. [45] and revised by Leung et al. [46], was used to assess the level of harsh discipline during childhood as perceived by adolescents. The psychological aggression and physical punishment subscales consisted of 16 items on a 7-point scale, with 1 indicating “never” and 7 indicating “almost every day”. Participants were asked to recall the frequency of experiencing two types of harsh discipline from their parents during their childhood. The mean score was calculated, with higher scores indicating higher levels of harsh discipline from parents. Previous research has shown that this questionnaire has good reliability and validity when used with Chinese adolescent populations [26]. The Cronbach’s α for the psychological aggression scale in this study was 0.86, and for the corporal punishment scale it was 0.96.

#### 2.2.2. Self-Compassion

The Self-Compassion Scale by Neff [47] was used to measure self-compassion in this study. The scale consists of 26 items and is composed of six subscales: self-kindness, common humanity, mindfulness, isolation, self-judgment, and over-identification. The first three subscales represent the positive aspects of self-compassion, while the last three subscales reflect the negative aspects of self-compassion (reverse scored). All items were rated on a 5-point scale (1 = strongly disagree, 5 = strongly agree). The Chinese version of the scale has been shown to have good reliability and structural validity in Chinese samples [48]. In the present study, the Cronbach’s α for the scale was 0.87.

#### 2.2.3. Aggressive Behavior

The present study was assessed using the aggression subscale of the Youth Risk Behavior Questionnaire (YRBS) developed by Brener et al. [49] and adapted by Zhou et al. [50]. The Chinese version of this scale has been demonstrated to have good reliability and construct validity in Chinese samples [51]. The aggressive behavior scale includes three items: “engaging in violent conflicts with others”, “getting into arguments with others”, and “engaging in self-harming behavior”. Participants rate their behavior on a 3-point scale (1 = never, 3 = almost always) based on the past six months. In this study, the Cronbach’s α values for adolescent aggressive behavior at T1, T2, and T3 were 0.77, 0.84, and 0.87, respectively.

#### 2.2.4. Socio-Economic Status

Participants evaluated their subjective socio-economic status (SES) on a scale from 1 to 10, with 10 representing the highest advantage in terms of money, education, and employment and 1 representing the most disadvantaged position (M = 4.65, SD = 1.40).

### 2.3. Procedure

First, the purpose, procedures, and instructions of the assessment were explained to the school leaders and teachers by psychologists. Written informed consent forms from the parents and adolescents were collected before conducting the online questionnaire assessment. Specifically, the adolescents were organized by their teachers to fill out the questionnaire in the school’s computer room. The instructions for the questionnaire clearly explained the need to answer the questions with as much focus as possible and emphasized that there were no right or wrong answers; they just needed to provide independent responses based on their actual experiences. The participants were informed that they could choose not to complete all the items if they felt uncomfortable or refused to answer. Additionally, the participants were assured that the collected data would be managed by the researchers and kept strictly confidential from their teachers. A unique ID number was assigned to each student for data matching purposes. Finally, ways to seek professional psychological help and advice were provided at the end of the questionnaire. All materials and procedures of this study were approved by the Institute Review Board (IRB) of Zhejiang Normal University; the ethical code is D2020009.

### 2.4. Data Analysis

Data Integrity: Little’s MCAR test [52] revealed that the missing data in this study were completely random (*χ*^2^ = 58.43, *df* = 48, *p* = 0.14). Therefore, this study utilized the expectation-maximization (EM)-based multiple imputation method to handle missing data [53,54].

In a preliminary step, the critical assumptions of the SEM were examined using SPSS 26.0. Firstly, the normality assumption was tested to determine whether each study variable approximated a normal distribution. The Shapiro–Wilk test values for childhood psychological abuse and physical punishment, adolescent self-compassion, and aggressive behavior at corresponding time points were all non-significant (*p* > 0.05). Additionally, the skewness and kurtosis values of all variables ranged from 1.08 to 2.34 (<3.29), indicating that the data were approximately normally distributed [55]. Secondly, the multicollinearity analysis results showed that the tolerance values for all predictor variables ranged from 0.48 to 0.57 (>0.1), and the variance inflation factor ranged from 1.05 to 1.75 (<10), indicating the absence of multicollinearity issues among the predictor variables [56]. Thirdly, the assumption of homoscedasticity was examined. The Levene’s Test of Equality of Variances showed no statistical differences in the variances of the outcome variables across groups (0.08 ≤ *p* ≤ 0.41), indicating that the assumption of equal variances was met. Based on these results, descriptive statistics and correlation analyses were performed using SPSS 26.0.

Subsequently, a structural equation model (SEM) was established and analyzed using Mplus 8.3 in three steps based on the research objectives. First, an unconditional latent growth model (LGM) was employed to examine the developmental trajectory of adolescent aggressive behavior [57]. The LGM extracted the intercept and slope when describing the trajectory of variable development. The intercept represented the initial level of variable development, with all factor loadings fixed at 1. The slope represented the rate of variable development. According to the requirements of the LGM, a linear trajectory was fitted in this study; thus, the factor loadings of the slope were fixed at 0, 1, and 2 [57]. Second, a conditional latent growth model was developed using data on childhood psychological aggression and corporal punishment at the first measurement to examine their direct predictive effects on the intercept and slope of adolescent aggressive behavior. At the same time, the influence of adolescent gender, age, and SES on the intercept and slope of aggressive behavior was controlled to reduce potential interference from these variables on the direct effects of childhood harsh discipline. Third, on the basis of the second step, the mediating effect of self-compassion between childhood psychological aggression and corporal punishment and the intercept and slope of adolescent aggressive behavior were further examined, and the significance of the mediation effect was verified using the bootstrap method.

## 3. Results

### 3.1. Common Methodological Biases

This study controlled and tested the issue of common method bias through three steps. Firstly, in terms of procedures, this study emphasized anonymity, confidentiality, and the use of data solely for scientific research during the data collection process. Additionally, reverse scoring was applied to certain items. Secondly, Harman’s single-factor test was conducted to examine the common method bias in the data. The results showed that 10 factors had eigenvalues greater than 1, and the total variance explained by the first factor was 22.08%, which was below the critical threshold of 40% [58]. Furthermore, this study aggregated the three items with the highest factor loadings from each scale as corresponding latent variable indicators [59]. The confirmatory factor analysis of the single-factor model indicated poor model fit: *χ*^2^ = 4206.36, *df* = 103, CFI = 0.63, TLI = 0.45, RMSEA = 0.23, and SRMR = 0.15. Therefore, there was no apparent common method bias in this study.

### 3.2. Descriptive Statistics

The descriptive statistics and Pearson correlation coefficients are presented in Table 1. The results showed that, both within waves and across waves, there were positive correlations between harsh discipline during childhood, psychological aggression, and adolescent aggressive behavior. Additionally, self-pity was negatively correlated with harsh discipline during childhood, corporal punishment, and adolescent aggressive behavior.

### 3.3. Development Changes of Adolescent Aggressive Behavior

Latent growth models (LGMs) were used to examine the development trajectory of adolescent aggressive behavior. The results from the sample of this study (as shown in Table 2) demonstrated that the model of the development trajectory of adolescent aggressive behavior fit well and exhibited a linear declining trend. Furthermore, there was a significant negative correlation between the initial level (intercept) and the rate of change (slope) in adolescent aggression.

### 3.4. Direct Effects of Psychological Aggression and Corporal Punishment during Childhood on the Initial Level and Rate of Development of Adolescent Aggressive Behavior

**Figure 1:**
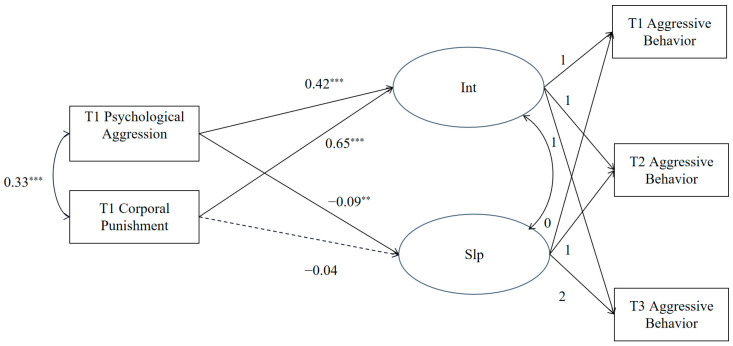
The direct effects model. Int = intercept; Slp = slope. ** *p* < 0.01, *** *p* < 0.001. The gender, age, and SES of adolescents were controlled in the model but not displayed in the figure for clarity of results.

Using psychological aggression and corporal punishment during childhood as predictor variables and adolescent aggressive behavior as the outcome variable, a conditional growth model was constructed (see Figure 1) to examine whether psychological aggression and corporal punishment could predict the initial level and rate of development of adolescent aggressive behavior. The results revealed that after controlling for adolescent gender, age, and SES, the conditional model fit well (*χ*^2^/*df* = 1.04, CFI = 0.999, TLI = 0.998, RMSEA = 0.01). Both psychological aggression and corporal punishment significantly positively predicted the intercept of adolescent aggressive behavior (*β* = 0.42, *p* < 0.001; *β* = 0.65, *p* < 0.001). Additionally, only childhood experience of psychological aggression directly slowed down the declining rate of adolescent aggressive behavior (*β* = −0.09, *p* = 0.018), and the direct predictive effect of childhood corporal punishment experiences on the rate of development of adolescent aggressive behavior was not significant (*β* = −0.04, *p* = 0.289). This suggests that the higher the level of psychological aggression and corporal punishment in childhood, the higher the initial level of adolescent aggression, and that the experience of psychological aggression in childhood impedes the rate of decline in adolescent aggressive behavior.

### 3.5. Mediating Role of Self-Compassion

**Figure 2:**
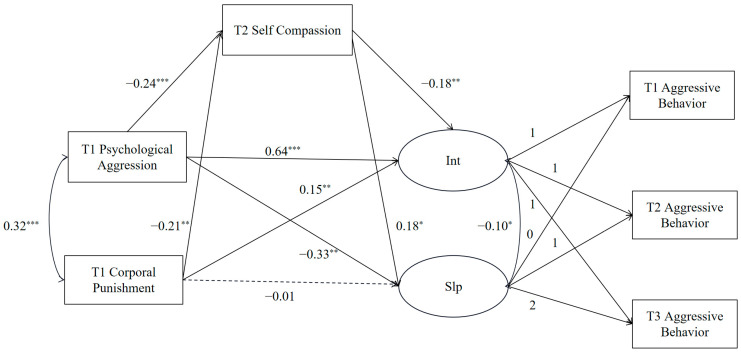
The indirect effects model. Int = intercept; Slp = slope. * *p* < 0.05, ** *p* < 0.01, *** *p* < 0.001. The gender, age, and SES of adolescents were controlled in the model but not displayed in the figure for clarity of results.

Based on the direct prediction model, the mediating effect of self-compassion on the relationship between childhood psychological aggression and corporal punishment and the development trajectory of adolescent aggressive behavior was examined. The results showed that the model fit well (*χ*^2^/*df* = 1.74, CFI = 0.994, TLI = 0.983, RMSEA = 0.03), and the final model is shown in Figure 2.

The direct paths between childhood psychological aggression and corporal punishment, adolescent self-compassion, and the initial level and rate of development of aggressive behavior are shown in Table 3. After controlling for gender, age, and SES, childhood psychological aggression and corporal punishment significantly and positively predicted initial levels of adolescent aggression and negatively predicted adolescent self-compassion. In addition, childhood psychological aggression negatively predicted the rate of decline in adolescent aggression, and adolescent self-compassion positively predicted the rate of decline in adolescent aggression.

Moreover, bootstrapping (repeated sampling 1000 times) was used to validate the mediating effect of self-compassion. The model included four indirect paths: (1) psychological aggression → self-compassion → intercept of adolescent aggressive behavior; (2) psychological aggression → self-compassion → slope of adolescent aggressive behavior; (3) corporal punishment → self-compassion → intercept of adolescent aggressive behavior; (4) corporal punishment → self-compassion → slope of adolescent aggressive behavior. The bootstrap results showed that all four indirect paths were significant (see Table 4), indicating that childhood psychological aggression and corporal punishment could indirectly influence the development of adolescent aggressive behavior, and self-compassion played a longitudinal mediating role in these association.

## 4. Discussion

Adolescence was a critical period of individual development, but the imbalance between physiological maturity and psychological development increased the risk of engagement in aggressive behaviors among adolescents [16]. However, previous studies predominantly focused on exploring the influencing factors and underlying mechanisms of adolescent aggressive behavior, lacking attention to the relationship between parenting styles during childhood and the developmental trajectory of adolescent aggression. Therefore, based on the social–cognitive information-processing models of aggression and general aggression models, the present study comprehensively explored the relationship between two typical childhood harsh disciplinary styles, psychological aggression and corporal punishment, and the initial level and developmental rate of adolescent aggression, and innovatively examined the mediating role of self-compassion in these relationships. The findings of this study provided additional evidence for theories related to harsh discipline, adolescent self-compassion, and the development of aggressive behavior. They also offered valuable insights for preventive and intervention programs aimed at improving adolescent aggressive behavior.

### 4.1. Developmental Changes in Adolescent Aggressive Behavior

Consistent with Hypothesis 1 and previous research suggesting a normative developmental trend of decreasing adolescent aggressive behavior with age [17,18], results from our sample in this study indicated an overall decline in adolescent aggressive behavior during the measurement period. This may be attributed to the gradual development of the brain cortex and the accumulation of social skills, enabling adolescents to consider the adverse consequences of aggressive behavior in the long term and to adopt more appropriate problem-solving strategies [60]. At the same time, the maturation of self-regulation abilities also helps adolescents to inhibit their destructive emotions and behavioral responses [61]. Additionally, participants in this study were first-year high-school students who were transitioning into high-school life at the time of the initial survey. This transition altered the familiar interpersonal and school environment for adolescents, bringing about more emotional fluctuations and changes in relationships, which increased the occurrence rate of aggressive behavior during this stage [62]. However, as adolescents gradually adapt to the new campus environment and establish new interpersonal relationships, their aggressive behavior also decreases.

Furthermore, there was a negative correlation between the initial level of adolescent aggressive behavior and the rate of decline in aggressive behavior throughout the measurement period. This suggests that the higher the initial level of adolescent aggressive behavior, the faster the rate of decline. Conversely, the lower the initial level of adolescent aggressive behavior, the slower the rate of decline. This finding supports previous research that suggests that the developmental differences in externalizing behavior among adolescents may gradually diminish with age [63,64]. It also indicates that aggressive behavior that does not slow down or even continues to increase during this stage may be atypical and portend significant future social adjustment problems for the individual [16], and it is necessary to focus on early risk factors that may undermine this normative developmental trend.

### 4.2. Direct Effects of Childhood Psychological Aggression and Corporal Punishment on the Developmental Changes of Adolescent Aggressive Behavior

This study found that, after controlling for adolescent gender, age, and SES, childhood experiences of psychological aggression and corporal punishment positively predicted the initial levels of adolescent aggressive behavior, consistent with Hypothesis 2 and previous research findings [27,30], indicating that harsh discipline during childhood may have profound negative effects on adolescents. According to the social–cognitive information-processing model of aggression, a conflict-ridden and harsh parenting environment reinforces children to use similar aggressive strategies to deal with problems [9]. Specifically, during early life, children learn acceptable behavioral standards and the consequences of not complying with these standards through interactions with caregivers. The more frequently parents restrict and regulate children’s behavior through verbal insults, blame, and corporal punishment, the more likely children perceive aggressive behavior as a reasonable and effective problem-solving strategy. When they feel dissatisfied with themselves or others in the future, they tend to apply this strategy to control their emotions or the behavior of others [65].

Interestingly, the results also found that childhood psychological aggression directly slowed down the rate of decline in adolescent aggressive behavior, but the direct predictive effect of childhood corporal punishment was not significant, partially inconsistent with our Hypothesis 3. The reason for this discrepancy may be that psychological aggression tends to cause more covert and enduring psychological injuries to adolescents compared to corporal punishment [66], whereas corporal punishment is characterized by immediate physical pain. Thus, while adolescents who experience childhood corporal punishment show higher levels of aggressive behavior, they still experience a gradual decline in aggression over time. In contrast, negative or derogatory comments from parents during childhood may lead children to have persistent negative evaluations of themselves and the external environment, hindering their acquisition of adaptive problem-solving strategies in adolescence and making it difficult for them to manage their aggressive behavior in a more positive manner. These findings not only support the social–cognitive information-processing model of aggression and previous research findings [31,32], but also further deepen the understanding of the dynamic relationship between different forms of harsh discipline during childhood and the developmental changes in adolescent aggressive behavior.

### 4.3. Mediating Role of Self-Compassion

This study also examined the longitudinal mediating role of self-compassion in the relationship between psychological aggression and corporal punishment during childhood and the development of adolescent aggressive behavior. Consistent with Hypotheses 4 and 5, the results of this study indicated that all four indirect paths were significant. This suggests that besides direct predictions, psychological aggression and corporal punishment during childhood also have indirect effects on the development of adolescent aggressive behavior through other pathways. This is in line with the general aggression model and the main findings of previous domestic and international research [13,41,67]. The general aggression model supports the idea that the influence of distal developmental environments on the outcomes of individual aggressive behavior may be mediated through shaping individual internal states [34]. In this study, self-compassion was identified as an important proximal factor that mediates the impact of negative parenting practices on the development of adolescent aggressive behavior.

On one hand, we found that psychological aggression and corporal punishment during childhood hinder the development of self-compassion in adolescents. When children are subjected to long-term criticism, blame, and physical punishment from their parents, they may internalize these negative messages and perceive themselves as unworthy of sympathy and care. Moreover, adolescence is a critical period for the formation of self-concept, and adolescents during this period may exaggerate their negative childhood experiences and incorporate them into their self-evaluation, resulting in negative self-awareness [43,68]. In fact, previous cross-sectional studies have shown that harsh parenting practices lead to the formation of negative self-cognitive schemas in adolescents, hindering the development of self-compassion [69].

On the other hand, harsh discipline during childhood, as a form of psychological aggression and corporal punishment, can further impact the initial level and rate of change of adolescent aggressive behavior. Self-compassion, as a positive cognitive and emotional regulation strategy [67], can help adolescents view setbacks and failures in life more objectively and tend to see their own experiences as part of a shared experience with other adolescents. Conversely, adolescents with low self-compassion may perceive their own suffering as incomprehensible to others and view the world as unjust, thus being more likely to adopt aggression as a strategy for managing emotions and interpersonal problems [70]. Additionally, self-compassion can predict the rate of decline in adolescent aggressive behavior during the tracking period. Adolescents who have experienced more psychological aggression and corporal punishment during childhood may have lower levels of self-compassion. These adolescents find it more difficult to treat themselves and others with understanding and kindness [43] and often exhibit higher levels of anger and lower levels of forgiveness [71], which hinders their ability to adopt more mature defense strategies and coping mechanisms when faced with emotional distress and interpersonal conflict, ultimately leading to a slower decline in adolescent aggressive behavior.

These findings suggest that childhood psychological aggression and corporal punishment may not only directly influence the initial level and rate of change of adolescent aggressive behavior but also enhance adolescent aggression by weakening their self-compassion, impeding the normative development of adolescent aggressive behavior. It is worth noting that adolescence is also an important period for self-concept and personality development [72]. Therefore, prevention and intervention measures aimed at reducing adolescent aggressive behavior may consider cultivating self-compassion as a promising strategy.

## 5. Limitations and Implications

There are some limitations in this study that need to be improved in future research. Firstly, the experiences of harsh discipline during childhood, self-compassion, and aggressive behavior in this study were all self-reported by adolescents. Although we ensured anonymity during the data collection process to enhance the truthfulness of the results, it is still difficult to avoid the problem of subjective bias that may arise in questionnaire studies. Future research should consider using multiple informant reports or combining various assessment methods to obtain more objective and diverse sources of data. Secondly, the participants in this study were all high-school students from eastern provinces in China, which may limit the generalizability of the findings to other populations. This limitation can be addressed by increasing sample diversity. Thirdly, although this study used an LGM approach to examine the linear declining trend of adolescent aggressive behavior, the limited number of follow-up assessments prevented explorations of possible curvilinear growth trends. Future research can further explore the developmental trajectory of the secondary growth of the variables of interest by extending the tracking time and increasing the number of tracking times. Lastly, the research question of this study can be further expanded and deepened. For example, this study only examined the overall developmental levels and rates of change of adolescent aggressive behavior. Future research could comprehensively consider the developmental characteristics of different types of aggressive behaviors, such as physical aggression, verbal aggression, and indirect aggression, and systematically explore the similarities and differences in the impacts of harsh discipline during childhood on various aspects of aggressive behavior. In addition, self-compassion and compassion towards others may play different roles in the relationship between parenting styles and the development of adolescent social behavior [73]. Future research can delve into this topic in more detail.

Despite these limitations, this study has important theoretical and practical implications. From a theoretical perspective, the current study systematically examined the unique influences of psychological aggression and corporal punishment, two representative forms of harsh discipline during childhood, on the development of adolescent aggressive behavior through three waves of assessments at six-month intervals and revealed the mediating role of self-compassion. These findings deepen the understanding of the mechanisms linking negative parenting experiences during childhood with the development of aggressive behavior in adolescents, providing a new perspective on how adverse childhood experiences relate to future aggressive behavior. From a practical perspective, this study may provide theoretical guidance for the development of more targeted prevention and intervention efforts to address adolescent aggressive behavior in practice, especially among adolescents who have experienced harsh discipline. For example, this study found that self-compassion is an important mediator linking childhood psychological aggression and corporal punishment to the initial level and rate of change in adolescent aggressive behavior, suggesting that enhancing self-compassion may be an effective way to alleviate adolescent aggression [36]. In addition, the impact of harsh discipline during childhood on the development and changes in adolescent aggressive behavior suggests that parents adopting warm and positive parenting styles rather than strict discipline and negative punishment may be more beneficial for the social development of children and adolescents.

## Figures and Tables

**Table 1:** Descriptive statistics and correlations among study variables. (*N* = 1214.)

	*M* ± *SD*	1	2	3	4	5	6	7	8	9
1. T1 PA	2.65 ± 0.92	1								
2. T2 CP	1.69 ± 1.01	0.65 ***	1							
3. T2 SC	3.18 ± 0.40	−0.13 ***	−0.12 ***	1						
4. T1 AB	1.41 ± 0.43	0.15 ***	0.19 ***	−0.23 ***	1					
5. T2 AB	1.35 ± 0.40	0.16 ***	0.17 ***	−0.24 ***	0.32 ***	1				
6. T3 AB	1.31 ± 0.39	0.15 ***	0.17 ***	−0.17 ***	0.30 ***	0.26 ***	1			
7. Gender	-	0.03	−0.07 *	0.01	−0.15 **	−0.12 **	−0.09 **	1		
8. Age	15.46 ± 0.71	−0.01	−0.01	0.04	0.02	0.02	0.03	−0.01	1	
9. SES	4.65 ± 1.40	−0.04	−0.01	0.04	−0.03	−0.07 *	−0.03	−0.05	0.04	1

Note. PA = psychological aggression; CP = corporal punishment; SC = self-compassion; AB = aggressive behavior. * *p* < 0.05, ** *p* < 0.01, *** *p* < 0.001.

**Table 2:** Model fit indices with intercepts and slopes for potential growth in adolescent aggressive behavior.

	*χ*^2^/*df*	RMSEA	CFI	TLI	SRMR	Means		r
						Int	Slp	
Aggressive Behavior	0.76	0.00	1.00	1.00	0.01	1.40 ***	−0.05 ***	−0.10 **

Note. ** *p* < 0.01, *** *p* < 0.001.

**Table 3:** Direct paths in the vertical intermediation model.

Direct Paths	B	SE	*p*
PA → SC	−0.24	0.04	0.000
PA → Int	0.64	0.07	0.000
PA → Slp	−0.33	0.08	0.002
CP → SC	−0.21	0.03	0.000
CP → Int	0.15	0.05	0.003
CP → Slp	−0.01	0.09	0.942
SC → Int	−0.18	0.04	0.000
SC → Slp	0.18	0.08	0.015

Note. PA = psychological aggression; CP = corporal punishment; SC = self-compassion.

**Table 4:** Indirect paths in vertical intermediation models.

Indirect Paths	B	*SE*	*p*	Standardized 95% CI	
				Low	High
PA → SC → Int	0.10	0.01	0.000	0.063	0.173
CP → SC → Int	0.10	0.02	0.000	0.057	0.169
PA → SC → Slp	−0.11	0.01	0.002	−0.197	−0.020
CP → SC → Slp	−0.10	0.01	0.004	−0.196	−0.038

Note. PA = psychological aggression; CP = corporal punishment; SC = self-compassion.

## Data Availability

The data supporting this study’s findings are available from the corresponding author upon reasonable request.

## References

[1] Connor D.F. (2004). Aggression and Antisocial Behavior in Children and Adolescents: Research and Treatment.

[2] World Health Organization Youth Violence. https://www.who.int/multi-media/details/youth-violence.

[3] Esposito C., Spadari E.M., Caravita S.C., Bacchini D. (2022). Profiles of community violence exposure, moral disengagement, and bullying perpetration: Evidence from a sample of Italian adolescents. J. Interpers. Violence.

[4] Irwin V., Zhang J., Wang X., Hein S., Wang K., Roberts A., Purcell S. Report on the Condition of Education 2021 (NCES 2021-144). U.S. Department of Education, National Center for Education Statistics. https://nces.ed.gov/pubsearch/pubsinfo.asp?pubid=2021144.

[5] Ettekal I., Ladd G.W. (2015). Developmental pathways from childhood aggression-disruptiveness, chronic peer rejection, and deviant friendships to early-adolescent rule breaking. Child Dev..

[6] Vuoksimaa E., Rose R.J., Pulkkinen L., Palviainen T., Rimfeld K., Lundström S., Kaprio J. (2021). Higher aggression is related to poorer academic performance in compulsory education. J. Child Psychol. Psychiatry.

[7] Polanin J.R., Espelage D.L., Grotpeter J.K., Spinney E., Ingram K.M., Valido A., El Sheikh A., Torgal C., Robinson L. (2021). A meta-analysis of longitudinal partial correlations between school violence and mental health, school performance, and criminal or delinquent acts. Psychol. Bull..

[8] Taylor L.K., Merrilees C.E., Goeke-Morey M.C., Shirlow P., Cummings E.M. (2016). Trajectories of adolescent aggression and family cohesion: The potential to perpetuate or ameliorate political conflict. J. Clin. Child Adolesc. Psychol..

[9] Pakaslahti L. (2000). Children’s and adolescents’ aggressive behavior in context: The development and application of aggressive problem-solving strategies. Aggress. Violent Behav..

[10] Kelly R.J., Erath S.A., Martin-Piñón O., El-Sheikh M. (2021). Longitudinal relations between parents’ sleep problems and harsh parenting. J. Fam. Psychol..

[11] Wang M.T., Kenny S. (2014). Longitudinal links between fathers’ and mothers’ harsh verbal discipline and adolescents’ conduct problems and depressive symptoms. Child Dev..

[12] He N., Xiang Y. (2021). How child maltreatment impacts internalized/externalized aggression among Chinese adolescents from perspectives of social comparison and the general aggression model. Child Abus. Negl..

[13] Zhang C., Zhang Q., Wang S., Xu W. (2023). Childhood trauma and aggression among Chinese college students: The mediation of self-compassion and moderation of left-behind experience. Psychol. Trauma Theory Res. Pract. Policy.

[14] Casey B.J., Jones R.M., Levita L., Libby V., Pattwell S.S., Ruberry E.J., Soliman F., Somerville L.H. (2010). The storm and stress of adolescence: Insights from human imaging and mouse genetics. Dev Psychobiol..

[15] Collado A., Felton J.W., MacPherson L., Lejuez C.W. (2014). Longitudinal trajectories of sensation seeking, risk taking propensity, and impulsivity across early to middle adolescence. Addict. Behav..

[16] Lickley R.A., Sebastian C.L. (2018). The neural basis of reactive aggression and its development in adolescence. Psychol. Crime Law.

[17] Yuan C., Shao A., Chen X., Xin T., Wang L., Bian Y. (2014). Developmental trajectory and gender differences in Chinese adolescents’ physical and relational aggression: An analysis using the latent class growth model. J. Aggress. Confl. Peace Res..

[18] Zuffianò A., Colasante T., Buchmann M., Malti T. (2018). The codevelopment of sympathy and overt aggression from middle childhood to early adolescence. Dev. Psychol..

[19] Cleverley K., Szatmari P., Vaillancourt T., Boyle M., Lipman E. (2012). Developmental trajectories of physical and indirect aggression from late childhood to adolescence: Sex differences and outcomes in emerging adulthood. J. Am. Acad. Child Adolesc. Psychiatry.

[20] Coyne S.M., Stockdale L.A., Warburton W., Gentile D.A., Yang C., Merrill B.M. (2020). Pathological video game symptoms from adolescence to emerging adulthood: A 6-year longitudinal study of trajectories, predictors, and outcomes. Dev. Psychol..

[21] Duggins S.D., Kuperminc G.P., Henrich C.C., Smalls-Glover C., Perilla J.L. (2016). Aggression among adolescent victims of school bullying: Protective roles of family and school connectedness. Psychol. Violence.

[22] Karriker-Jaffe K.J., Foshee V.A., Ennett S.T., Suchindran C. (2008). The development of aggression during adolescence: Sex differences in trajectories of physical and social aggression among youth in rural areas. J. Abnorm. Child Psychol..

[23] Huffman L.G., Oshri A., Caughy M. (2020). An autonomic nervous system context of harsh parenting and youth aggression versus delinquency. Biol. Psychol..

[24] Kingsbury M., Sucha E., Manion I., Gilman S.E., Colman I. (2020). Adolescent mental health following exposure to positive and harsh parenting in childhood. Can. J. Psychiatry.

[25] Liu L., Wang M. (2015). Parenting stress and children’s problem behavior in China: The mediating role of parental psychological aggression. J. Fam. Psychol..

[26] Wang M., Liu L. (2018). Reciprocal relations between harsh discipline and children’s externalizing behavior in China: A 5-year longitudinal study. Child Dev..

[27] Cui G., Lan X. (2020). The associations of parental harsh discipline, adolescents’ gender, and grit profiles with aggressive behavior among Chinese early adolescents. Front. Psychol..

[28] MacKenzie M.J., Nicklas E., Brooks-Gunn J., Waldfogel J. (2015). Spanking and children’s externalizing behavior across the first decade of life: Evidence for transactional processes. J. Youth Adolesc..

[29] Hibbard R., Barlow J., MacMillan H., Christian C.W., Crawford-Jakubiak J.E., Flaherty E.G., Leventhal J.M., Lukefahr J.L., Sege R.D., Committee on Child Abuse and Neglect and American Academy of Child and Adolescent Psychiatry, Child Maltreatment and Violence Committee (2012). Psychological maltreatment. Pediatrics.

[30] Gómez-Ortiz O., Romera E.M., Ortega-Ruiz R. (2016). Parenting styles and bullying. The mediating role of parental psychological aggression and physical punishment. Child Abus. Negl..

[31] Prinzie P., Onghena P., Hellinckx W. (2006). A cohort-sequential multivariate latent growth curve analysis of normative CBCL aggressive and delinquent problem behavior: Associations with harsh discipline and gender. Int. J. Behav. Dev..

[32] Baydar N., Akcinar B. (2018). Reciprocal relations between the trajectories of mothers’ harsh discipline, responsiveness and aggression in early childhood. J. Abnorm. Child Psychol..

[33] Xie H., Drabick D.A., Chen D. (2011). Developmental trajectories of aggression from late childhood through adolescence: Similarities and differences across gender. Aggress. Behavior..

[34] Allen J.J., Anderson C.A., Bushman B.J. (2018). The general aggression model. Curr. Opin. Psychol..

[35] DeWall C.N., Anderson C.A., Bushman B.J. (2011). The general aggression model: Theoretical extensions to violence. Psychol. Violence.

[36] Neff K.D. (2023). Self-compassion: Theory, method, research, and intervention. Annu. Rev. Psychol..

[37] Bluth K., Gaylord S.A., Campo R.A., Mullarkey M.C., Hobbs L. (2016). Making friends with yourself: A mixed methods pilot study of a mindful self-compassion program for adolescents. Mindfulness.

[38] Raque T.L., Ziemer K., Jackson J., Finlay-Jones A., Bluth K., Neff K. (2023). Attachment and Self-Compassion: Associations Across the Lifespan. Handbook of Self-Compassion.

[39] Pepping C.A., Davis P.J., O’Donovan A., Pal J. (2015). Individual differences in self-compassion: The role of attachment and experiences of parenting in childhood. Self Ident..

[40] Chi X., Jiang W., Guo T., Hall D.L., Luberto C.M., Zou L. (2022). Relationship between adverse childhood experiences and anxiety symptoms among Chinese adolescents: The role of self-compassion and social support. Curr. Psychol..

[41] Miyagawa Y., Taniguchi J. (2022). Sticking fewer (or more) pins into a doll? The role of self-compassion in the relations between interpersonal goals and aggression. Motiv. Emot..

[42] Neff K.D., Beretvas S.N. (2013). The role of self-compassion in romantic relationships. Self Ident..

[43] Neff K.D., McGehee P. (2010). Self-compassion and psychological resilience among adolescents and young adults. Self Ident..

[44] Fatima S., Sheikh H. (2014). Socioeconomic status and adolescent aggression: The role of executive functioning as a mediator. Am. J. Psychol..

[45] Straus M.A., Hamby S.L., Finkelhor D., Moore D.W., Runyan D. (1998). Identification of child maltreatment with the Parent-Child Conflict Tactics Scales: Development and psychometric data for a national sample of American parents. Child Abus. Negl..

[46] Leung P.W., Wong W.C., Chen W.Q., Tang C.S. (2008). Prevalence and determinants of child maltreatment among high school students in Southern China: A large scale school based survey. Child Adolesc. Psychiatry Ment. Health.

[47] Neff K.D. (2003). Development and validation of a scale to measure self-compassion. Self Ident..

[48] Wang D., Xie R., Ding W., Yuan Z., Kayani S., Li W. (2022). Bidirectional longitudinal relationships between parents’ marital satisfaction, parenting stress, and self-compassion in China. Fam. Process.

[49] Brener N.D., Collins J.L., Kann L., Warren C.W., Williams B.I. (1995). Reliability of the youth risk behavior survey questionnaire. Am. J. Epidemiol..

[50] Zhou X., Wu X. (2015). Positive Cognition Moderate the Relationship between Posttraumatic Stress Disorder on Sleep Problems among Children Survivors after Wenchuan Earthquake. Stud. Psychol. Behav..

[51] Zhou X., Wu X., Chen Q., Zhen R. (2017). Why did adolescents have sleep problems after earthquakes? Understanding the role of traumatic exposure, fear, and PTSD. Scand. J. Psychol..

[52] Li C. (2013). Little’s test of missing completely at random. Stata J..

[53] Bennett D.A. (2001). How can I deal with missing data in my study?. Aust. N. Z. J. Public Health.

[54] Schlomer G.L., Bauman S., Card N.A. (2010). Best practices for missing data management in counseling psychology. J. Couns. Psychol..

[55] Field A. (2013). Discovering Statistics Using SPSS.

[56] Wen Z.L., Huang B.B., Tang D.D. (2018). Preliminary work for modeling questionnaire data. J. Psychol. Sci..

[57] Bollen K.A., Curran P.J. (2006). Latent Curve Models: A Structural Equation Perspective.

[58] Podsakoff P.M., MacKenzie S.B., Lee J.Y., Podsakoff N.P. (2003). Common method biases in behavioral research: A critical review of the literature and recommended remedies. J. Appl. Psychol..

[59] Wu Y., Wen Z.L. (2011). Item parceling strategies in structural equation modeling. Adv. Psychol. Sci..

[60] Chang T.T., Metcalfe A.W., Padmanabhan A., Chen T., Menon V. (2016). Heterogeneous and nonlinear development of human posterior parietal cortex function. NeuroImage.

[61] Farley J.P., Kim-Spoon J. (2014). The development of adolescent self-regulation: Reviewing the role of parent, peer, friend, and romantic relationships. J. Adolesc..

[62] Williford A.P., Brisson D., Bender K.A., Jenson J.M., Forrest-Bank S. (2011). Patterns of aggressive behavior and peer victimization from childhood to early adolescence: A latent class analysis. J. Youth Adolesc..

[63] Bongers I.L., Koot H.M., van der Ende J., Verhulst F.C. (2003). The normative development of child and adolescent problem behavior. J. Abnorm. Psychol..

[64] Johnson M.K., Crosnoe R., Elder G.H. (2011). Insights on adolescence from a life course perspective. J. Res. Adolesc..

[65] Van Heel M., Bijttebier P., Colpin H., Goossens L., Van Den Noortgate W., Verschueren K., Van Leeuwen K. (2019). Investigating the interplay between adolescent personality, parental control, and externalizing problem behavior across adolescence. J. Res. Personal..

[66] Tian W., Wang F., Wang M. (2023). Parental Marital Quality and Children’s Depression in China: The Different Mediating Roles of Parental Psychological Aggression and Corporal Punishment. J. Fam. Violence.

[67] Tao J., He K., Xu J. (2021). The mediating effect of self-compassion on the relationship between childhood maltreatment and depression. J. Affect. Disord..

[68] Alberts A., Elkind D., Ginsberg S. (2007). The personal fable and risk-taking in early adolescence. J. Youth Adolesc..

[69] Liu Q.Q., Hu Y.T. (2023). Self-compassion mediates and moderates the association between harsh parenting and depressive symptoms in Chinese adolescent. Curr. Psychol..

[70] Fresnics A., Borders A. (2017). Angry rumination mediates the unique associations between self-compassion and anger and aggression. Mindfulness.

[71] Wu Q., Chi P., Zeng X., Lin X., Du H. (2019). Roles of anger and rumination in the relationship between self-compassion and forgiveness. Mindfulness.

[72] Telzer E.H., Dai J., Capella J.J., Sobrino M., Garrett S.L. (2022). Challenging stereotypes of teens: Reframing adolescence as window of opportunity. Am. Psychol..

[73] Marshall S.L., Ciarrochi J., Parker P.D., Sahdra B.K. (2020). Is self-compassion selfish? The development of self-compassion, empathy, and prosocial behavior in adolescence. J. Res. Adolesc..

